# A Patient with Cryoglobulinemic Membranoproliferative GN (MPGN) Who Survived COVID-19 Disease: Case Presentation and Current Data of COVID-19 Infection in Dialysis and Transplanted Patients in Greece

**DOI:** 10.3390/medicina56070355

**Published:** 2020-07-17

**Authors:** Smaragdi Marinaki, Stathis Tsiakas, Chrysanthi Skalioti, Panayiota Lourida, Aikaterini Argyraki, Konstantinos Grigorakos, Ioannis Boletis

**Affiliations:** 1 Clinic of Nephrology and Renal Transplantation, Medical School, Laiko Hospital, National and Kapodistrian University of Athens, 11527 Athens, Greece; smaragdimarinaki@yahoo.com (S.M.); stathis.tsiakas@gmail.com (S.T.); laikneph@laiko.gr (I.B.); 2 Infectious Diseases Clinic A, Sotiria Chest Diseases Hospital, 11527 Athens, Greece; giotalourida@gmail.com (P.L.); katrin.argyraki@gmail.com (A.A.); 3 Independent Researcher, 12 Protopappa Avenue, 16345 Athens, Greece; gk_pediatr@yahoo.gr

**Keywords:** COVID-19, pneumonia, glomerulonephritis, immunosuppression, transplantation, Renal Replacement Therapy (RRT)

## Abstract

The evolving pandemic of Coronavirus Disease 2019 has posed a substantial health risk worldwide. However, there is a paucity of data regarding the clinical course and the therapeutic management of patients with chronic kidney disease and COVID-19 infection. To date, most evidence has come from renal transplantation, with about 45 patients reported thus far, and the current data from the ERA-EDTA (ERACODA) registry for transplanted patients and patients on Renal Replacement Therapy (RRT); as for those with glomerular diseases, data are lacking. Herein, we report the case of a 62-year-old patient with severe membranoproliferative glomerulonephritis who had been receiving a high burden of immunosuppression until four months before the COVID-19 infection. He developed severe disease with acute respiratory failure requiring mechanical ventilation. After treatment with hydroxychloroquine and azithromycin, despite his low chances, he gradually recovered and survived. To the best of our knowledge, this is one of the few reported patients with glomerulonephritis who had COVID-19 Besides our single case with glomerulonephritis early during the disease outbreak, the very low prevalence of COVID-19 infection in the country’s transplant recipients (0.038%) and dialysis patients (0.24%) reflects the impact of the rapid implementation of social distancing rules as well as of preventive measures for disease control in the hospitals and dialysis units in our country.

## 1. Introduction

We are currently in the midst of the third epidemic of coronaviruses in the last 20 years. The new virus SARS-CoV-2 belongs to the β-coronavirus cluster, which also comprises the viruses that cause Severe Acute Respiratory Syndrome (SARS) and Middle East Respiratory Syndrome (MERS), emerging in 2003 and2012, respectively [1].

The new virus causes SARS-CoV-2-related disease 2019 (COVID-19). It started in December 2019 in the city of Wuhan in central China and spread rapidly across the world, reaching proportions of a “global pandemic” by 15 March 2020 [2].

Along with other European countries, Greece has been affected, with the first confirmed case being identified on 26 February 2020. 

In our Clinic for Nephrology and Renal Transplantation of Laiko Hospital of Athens, a tertiary hospital with an extensive kidney transplantation program, a total of 1450 transplanted patients are monitored every year. As the clinic further includes a Reference Center for Glomerular Diseases, an additional 520 patients with glomerular diseases are also under follow-up. Furthermore, we have a Hemodialysis Unit with 55 patients on Renal Replacement Therapy (RRT). It is noteworthy that, since the disease outbreak in our country and for the entire period of the past three months, our department had no kidney transplant recipients or RRT patientswith COVID-19 infection and only this single patient with glomerular disease with COVID-19 infection. 

## 2. Case Report

A 62-year-old male patient with a history of Chronic Lymphocytic Leukemia (CLL) (RAI stage 0) was initially evaluated in our department in July 2019 for nephrotic syndrome (Upr:12.7g/day) and impaired kidney function (Cr:1.98mg/dL). His kidney biopsy revealed features of cryoglobulinemic glomerulonephritis. Treatment with rituximab (four weekly doses of 375 mg/m^2^), cyclophosphamide (threemonthly iv doses of 500 mg/m^2^) and corticosteroids (prednisolone 0.75 mg/kg for threeweeks, followed by a slow taper) was initiated. The last infusion of cyclophosphamide was administered in October 2019. At his last follow-up in February 2020, sixmonths after therapy initiation, the patient had a creatinine level of 1.59 mg/dL with proteinuria 4.93 g/day while he was still receiving 6 mg of methylprednisolone per day. 

**Figure 1:**
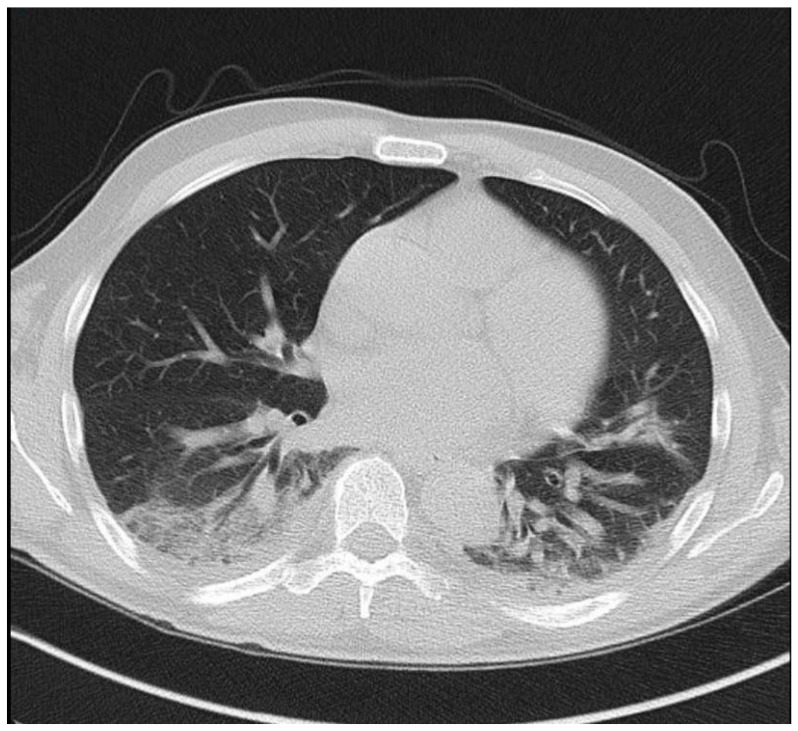
Multifocal ground glass density lesions were observed on a CT scan in the lower lobes bilaterally.

On 16 March, he presented to the emergency department with high fever and dry cough. He reported no contact with a coronavirus-infected patient or travel history. His symptoms started fivedays before presentation and rapidly deteriorated on the day of admission. On physical examination, he was tachypnoeic with SatO_2_: 93%. The throat swab sample for SARS-CoV-2 (RT-PCR) was positive, and the patient was transferred to a referral hospital for COVID-19. His blood tests revealed a white blood cell count of 5800 × 10^9^/L (neutrophils 58%, lymphocytes 36%), a C-reactive protein level of 65 mg/L (nr < 5) and slightly elevated d-dimers. A hepatic panel, LDH value and procalcitonin level were normal. A chest CTscan showed diffuse bilateral infiltrates (Figure 1). Besides respiratory support with oxygen therapy, treatment with hydroxychloroquine (200 mg bid) and ceftriaxone was initiated. The patient’s clinical status deteriorated rapidly, and, twodays later, he was transferred to the intensive care unit (ICU), where he was put on mechanical ventilation due to respiratory failure. Azithromycin (500 mg od for seven days) was added in the ICU. Remdesivir was not administered because of renal impairment (peak creatinine level of 2.8 mg/dL, corresponding to an eGFR of 23 mL/min/1.73 m^2^ by the CKD-EPI equation). The patient remained hemodynamically stable without vasopressors and maintained a satisfactory urine output despite a transient renal function deterioration. His respiratory function gradually improved, and he was discharged from the ICU after seven days and transferred to the rehabilitation unit. Meanwhile, a second CT scan on the 16th day of hospitalization showed a significant improvement of the lung lesions. After 25 days of hospitalization, he was discharged, and he remains in good clinical condition. His creatinine level has returned to 1.4 mg/dL.

## 3. Discussion

Very few patients on immunosuppressive treatment due to primary or secondary glomerular disease with confirmed SARS-CoV-2 infection have been reported thusfar [3]. Our patient with membranoproliferative glomerulonephritis (MPGN) survived after the development of severe pneumonia and acute respiratory failure requiring mechanical ventilation. Very little is known about COVID-19 infection in patients with glomerular diseases. These patients have been advised to respect social distancing rules since the early stages of the COVID-19 outbreak in all European countries. Most of the existing evidence comes from kidney transplantation.

Despite their increased vulnerability, the prevalence of COVID-19 virus infection has remained low in kidney transplant recipients, with reported rates varying from 1.6% to 3% among European countries [4,5] and the lowest reported rate being 0.33% [6]. This is due to the even stricter implementation of hygiene and social isolation measures relative to those in the general population and the compliance by the patients themselves. At the same time, according to the European Centre for Disease Prevention and Control, the prevalence of COVID-19 infection in the general population in the countries of the Eurozone is 0.29% [7].

### 3.1. Symptoms at Presentation

Our patient had the typical initial presentation of COVID-19 as in the general population, with fever and dry cough as the primary symptoms.

Most transplanted patients have also reported the typically described “flu-like” symptoms with fever, dry cough and malaise; only a minority have presented with diarrhea and abdominal pain [8].

### 3.2. Risk Factors

Our patient had several risk factors for severe COVID-19 disease: he was a middle-aged male with severe MPGN nephritis since 2019 with Chronic Kidney Disease (CKD) stage III (eGFR 46 mL/min, CKD-EPI) nephrotic-range (4.9 g/24 h) proteinuria and concomitant, albeit quiescent, CLL. He had received immunosuppression with the mAb Rituximab, a total of 3 g of cyclophosphamide and high-dose steroids 4.5 months before the COVID-19 infection.

### 3.3. Disease Course and Treatment of Immunocompromised Individuals

According to the European Centre for Disease Prevention and Control (ECDC), the majority of infected individuals (>80%) will run an indolent course with mild disease, another 15% will have a more severe form, and a minority, about 5%, will develop life-threatening disease [6]. A three-stage classification system that describes the three grades of increasing severity of COVID-19 disease was recently proposed by Siddiqi et al. [9]. According to this classification, our patient had severe illness (Stage IIb pneumonia with hypoxia), and, fortunately, he did not progress to Stage III, despite his comorbidities and immunocompromised status. Many questions about the behavior of COVID infection in immunocompromised individuals are still unanswered. Little is known about the exact disease course, while most evidence again comes from transplanted patients.

Immunosuppression undoubtedly places the patient into a high-risk group for acquiring as well as transmitting the virus [10]. Most agree that, as in our patient, the initial presentation does not differ from that in the general population [11].

Kidney transplant recipients who are infected with the COVID-19 virus have, as expected, a more severe disease and worse prognosis with higher rates of hospitalization and a need for mechanical ventilation support, as well as higher case fatality rates [5,6].

As for treatment, preventive measures are the same as those for the general population, with increased vigilance [12]. Most drugs used in other patients with COVID-19 virus infection may be used, with dose adjustments according to reduced renal function and consideration of their interactions with immunosuppressants.

Regarding the handling of immunosuppression, the general principles applicable to all severe, opportunistic or viral infections also apply for COVID-19 infection: most important is a reduction in the overall burden of immunosuppression. The first step is to discontinue immunosuppressive drugs that not only promote viral replication but also cause leukopenia, such as antimetabolites (mycophenolic acid and less often azathioprine), while, in more severe cases, calcineurin inhibitors (CNIs) can also be drastically reduced and/or discontinued. In any case, the decision is individualized by taking into account both the severity of the infection and the life threat against the consequence of potential graft loss.

The most recent review of COVID-19 infection in kidney transplant recipients includes 12 studies reporting on a total of 40 patients [13]. There was a broad variation in the time from renal transplantation, ranging from 1 month to 22 years, in baseline immunosuppression and in disease course and severity. Of the 40 reported patients, 18% developed life-threatening disease and eightdied (case fatality rate of 20%).

It must be emphasized, however, that most of the reported recipients were transplanted long before infection and were on low-dose maintenance immunosuppression, which was drastically reduced or discontinued during COVID-19 disease. This is a striking difference from patients with glomerular diseases, who might have received, as ours did, a high immunosuppressive load a short time before the infection. Our patient had received 3 g of cyclophosphamide and a cumulative methylprednisolone dose of 5 g a few months before the COVID infection. He also had received Rituximab six months before, and we assume that B-cells must have still been depleted [14]. Perhaps the patient’s previous good performance status, immediate diagnosis and prompt treatment initiation contributed to his favorable outcome.

The current opinion-based advice from the ERA-EDTA Immunonephrology Working Group for CKD patients with glomerulonephritis or vasculitis is to continue with immunosuppressive treatment in asymptomatic COVID-19 and reduce it cautiously, balancing the risk of relapse, in symptomatic patients. New-onset or relapsing vasculitis should be treated with immunosuppressive drugs, but, for new-onset glomerulonephritis, supportive care may be recommended as first-line treatment, and immunosuppression should be withheld and the anti-CD20 mAb Rituximab as maintenance therapy should be postponed if possible [15].

### 3.4. Epidemiology and Impact of Preventive Measures

In Greece, the first COVID-19 case was confirmed on 26 February 2020.

On 10 March with officially 8 cases/1 million population and 0 deaths, the Greek Government decided to close all educational structures and, on 13 March, to suspend the operation of leisure shops, museums, shopping malls, sports facilities and restaurants. On 16 March, all commercial stores were closed except for food and household businesses. On 23 March, significant restrictions were imposed on the movement of citizens [16].

**Figure 2:**
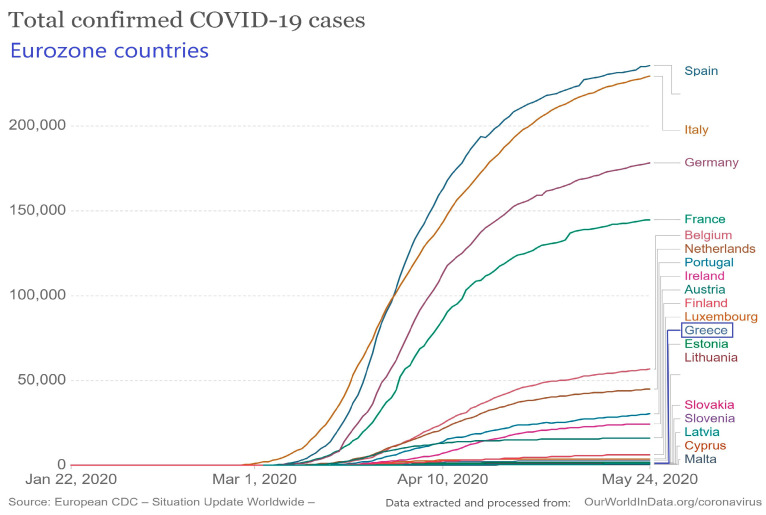
Total number of confirmed COVID-19 cases during the outbreak in Eurozone countries.

The total number of confirmed cases in the country as of 24 May is 2878 (55% men), the current number of intubated patients is 19 (68% men) and the death toll is 171 (71% men), with 100 patients having been discharged from the ICU (Figure 2) [17]. The average age of infected individuals is 48 years (range 0–102 years), while the average age of those who died is 76 years (range 35–102 years). In total, 153,963 clinical samples were tested, of which 4612 (3.0%) were positive for coronavirus (including more than one sample per person tested) [17].

The extensive and rapidly implemented measures taken by the Greek state are already reflected in the country’s epidemiological data.

**Figure 3:**
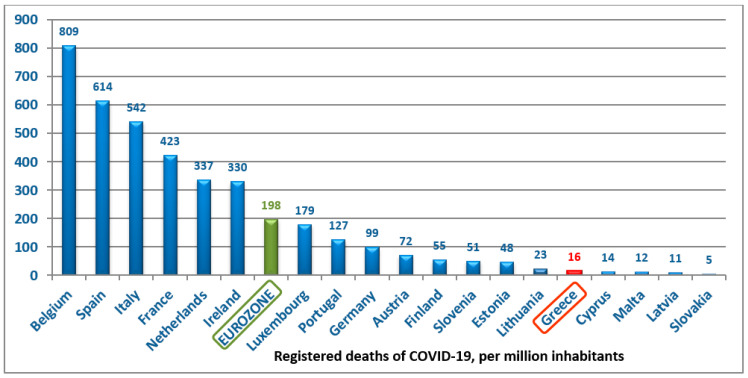
Registered deaths of COVID-19 per million population in Eurozone countries.

In relation to its population, Greece has 16 deaths per million population (1 M), which ranks the country in 70th place internationally (44.5/1 M, 215 countries), 39th in Europe (169.6/1 M, 50 countries) and 15th in the Eurozone (198/1 M, 19 countries) (Figure 3). The Case Fatality Rate (CFR: ratio between confirmed deaths and confirmed cases) in Greece is 5.9%, which is below the global average (6.2%) [18,19,20]. 

From the beginning of the COVID-19 outbreak in Greece, preventive measures were rapidly implemented in our hospital:

1. personnel training and application of infection control measures (hand hygiene, facial mask and gloves);
2. apredefined pathway in the Emergency Department for suspected cases, namelyscreening with NAT and isolation in a separate ward until the first COVID-19 PCR, after whichpatients with positive tests are transferred to one of the four referral hospitals of Athens, andpatients with negative tests are transferred to the normal ward under isolation until the second negative test; and
3. limitation of elective surgical procedures, including cessation of living donor transplantations.
  Guidelines were provided by the Hellenic Transplant Organisation on 15 March and the Hellenic Society of Nephrology on 17 March to all transplant centers and to all hospitals and dialysis units, respectively.
4. Patients undergoing maintenance dialysis were briefly evaluated for symptoms consistent with COVID-19 and had their body temperature measured before entering the Dialysis Unit. Handwashing and facemask use were mandatory upon entrance to the facility. Patients were separated by a distance of at least 1.5 m from each other and were required to keep their facemasks on during the entire dialysis session. No food or fluid intake was allowed while in the treatment area.

Patients were advised to stay home between dialysis sessions. In case of fever or respiratory symptoms, they were instructed to notify the Dialysis Unit before arriving to the facility. A divided area was organized for evaluating and testing these patients. Suspected cases were dialyzed in an isolation room in the dialysis ward. Confirmed cases were referred to a COVID-19 designated Dialysis Unit.

It is remarkable that, in the period from the beginning of the pandemic to date, from a total of 2567 patients with kidney transplantation all over the country, there has been only a single case of COVID-19 infection (prevalence of 0.038%) among the kidney transplant recipients who were tested.The patient had mild disease, was hospitalized and survived without developing life-threatening disease.

This clearly demonstrates the effectiveness of the social isolation measures and strict recommendations given by the nephrology units as well as the importance and efficacy of tele-medicine, which was successfully applied to monitor the population of kidney transplant recipients and patients with glomerular diseases during the “lock-down” period of the pandemic, while, since mid-May, a program of “controlled restart” of the regular outpatient clinics has been implemented.

Finally, a comparison between dialysis and transplantation during the COVID-19 era in our country comes out in favor of transplantation: to date, 28 confirmed cases of COVID-19 from a total population of 11,590 hemodialysis patients have been recorded in Greece (prevalence of 0.24%), and there have been sixdeaths (case fatality rate of 21%) (Table 1); data are lacking for patients with CKD Stages 1–4 [21]. This is in accordance with other countries where reported case fatality rates in hemodialysis patients are in the range of 20–30% [6].

## 4. Conclusions

Our sole COVID-19-infected patient with severe underlying glomerulonephritis survived, despite his very low chances. This is encouraging since nothing is more important than one saved life. Nevertheless, it is important to realize that, besides optimizing care for the single individual, preventive measures are essential for the control of the disease outbreak in the general population, which is eventually also reflected in very low infection rates in the vulnerable, immunocompromised patient populations of solid organ transplant recipients and glomerular disease patients on immunosuppressive treatment [5].

## Figures and Tables

**Table 1:** Epidemiology of COVID-19 in Greece, as of 24 May 2020.

	Total Population	Confirmed Cases	Prevalence	Confirmed Deaths	Mortality	Case Fatality Rate
Hemodialysispatients	11,590	28	0.24%	6	0.23%	21%
Transplantrecipients	2567	1	0.038%	0	0%	0%
General population	10,724,000	2878 ¹	0.026% ¹	171	0.0015%	5.9%

¹ The number of confirmed cases is lower than the number of actual cases because of testing limitations.
